# Highly stable semiconducting polymer nanoparticles for multi-responsive chemo/photothermal combined cancer therapy

**DOI:** 10.7150/thno.43090

**Published:** 2020-05-01

**Authors:** Yu Xu, Xue Zhai, Peng Su, Tianqi Liu, Luyao Zhou, Jingjing Zhang, Biqing Bao, Lianhui Wang

**Affiliations:** Key Laboratory for Organic Electronics and Information Displays & Jiangsu Key Laboratory for Biosensors, Institute of Advanced Materials (IAM), Jiangsu National Synergetic Innovation Center for Advanced Materials (SICAM), Nanjing University of Posts & Telecommunications, Nanjing 210023, China

**Keywords:** semiconducting polymer nanoparticles, semi-interpenetrating, multi-responsive, chemo/ photothermal therapy

## Abstract

**Rationale:** Structural stability and size controllability are critical issues to semiconducting polymer nanoparticles (SPNs), which currently show great potential for theranostic applications.

**Methods:** Herein, multi-responsive semiconducting polymer semi-interpenetrating nanoparticles (PDPP3T@PNIPAMAA IPNs) with highly stable structure and uniform size have been successfully designed by semi-interpenetrating technique.

**Results:** It is proposed for the first time that PDPP3T@PNIPAMAA IPNs were prepared with “reinforced concrete” particle structure, which is even resistant to organic solvent such as ethanol and THF. By adjusting the polymerization time, the obtained PDPP3T@PNIPAMAA IPNs exhibit uniform and controllable particle size with extremely low polydispersity index (~0.037) at 1 h of reaction time. The presence of pH/light/GSH multi-responsive semi-interpenetrating network in PDPP3T@PNIPAMAA IPNs dramatically increase their drug loading efficiency (92.64%), which is significantly higher than previously reported comparable SPNs-based drug delivery systems. Additionally, PDPP3T@PNIPAMAA-DOX IPNs further provide improved therapeutic efficacy by the combination of chemotherapy and photothermal therapy with controllably regulated release of doxorubicin (DOX). *In vitro* and *in vivo* results indicate that PDPP3T@PNIPAMAA-DOX IPNs are able to release drugs at controlled rate by pH/light/GSH regulation and offer PAI-guided chemo/photothermal combined therapy with excellent therapeutic efficacy.

**Conclusions:** The semi-interpenetrating network method may be generally extended for the preparation of a wide range of organic polymer nanoparticles to achieve ultrahigh structural stability, precise particle size controllability and excellent drug loading capacity.

## Introduction

Semiconducting polymer nanoparticles (SPNs) have found tremendous success in recent years as promising theranostic agent owing to their good optical properties, high photostability and outstanding biocompatibility 1-5. Up to now, SPNs have been diversified for biological sensing, drug delivery, photoacoustic imaging (PAI), as well as photothermal and photodynamic therapy. SPNs are composed of organic constituents with low cytotoxicity and thus naturally overcome the long term toxicity issue caused by inorganic nanoparticles 6-9. Despite the progress of SPNs in nanomedicine in recent years, fabrication techniques for SPNs with structurally stability and multi-functionality are still in its infancy and confronted with great challenges.

The synthetic techniques for making semiconducting polymer nanoparticles are generally limited to emulsion technique and nanoprecipitation method 10-13. However, these techniques offer poor control of SPNs size and produce SPNs with different particle size 14. Furthermore, to afford SPNs with surface functional groups, amphiphilic polymer or phospholipid are required to encapsulate SPNs surface 15-18, which however are not inherently stable and suffer from the potential dissociation issues. To achieve structural stability, the synthesis and self-assembly of poly(ethylene glycol) (PEG) grafting SPNs have recently been reported 19, 20. However, the synthesis of semiconducting polymer amphiphiles is relatively complicated and the functionalization is specifically designed for each SPNs. Therefore, new strategies to develop SPNs with stable structure and controllable sizes are desired but still a challenge.

Recently, multi-stimuli sensitive (e.g., pH changes, temperature, redox or light) nanoagents for controlled chemo/photothermal synergistic therapy have attracted considerable attention due to their additionally enhanced anticancer efficacy and reduced side effects. Some stimuli-responsive amphiphilic polymers have been used to coprecipitate with semiconducting polymer to fabricate SPNs-based drug delivery systems 21, 22. However, most of them do not have multi-stimuli responsive properties and show limited drug-loading efficiency (<30%). Furthermore, amphiphilic polymer could potentially escape from SPNs, which pose changes of their optical properties and also cause poor stimuli-responsiveness of SPNs 23.

**Figure 1:**
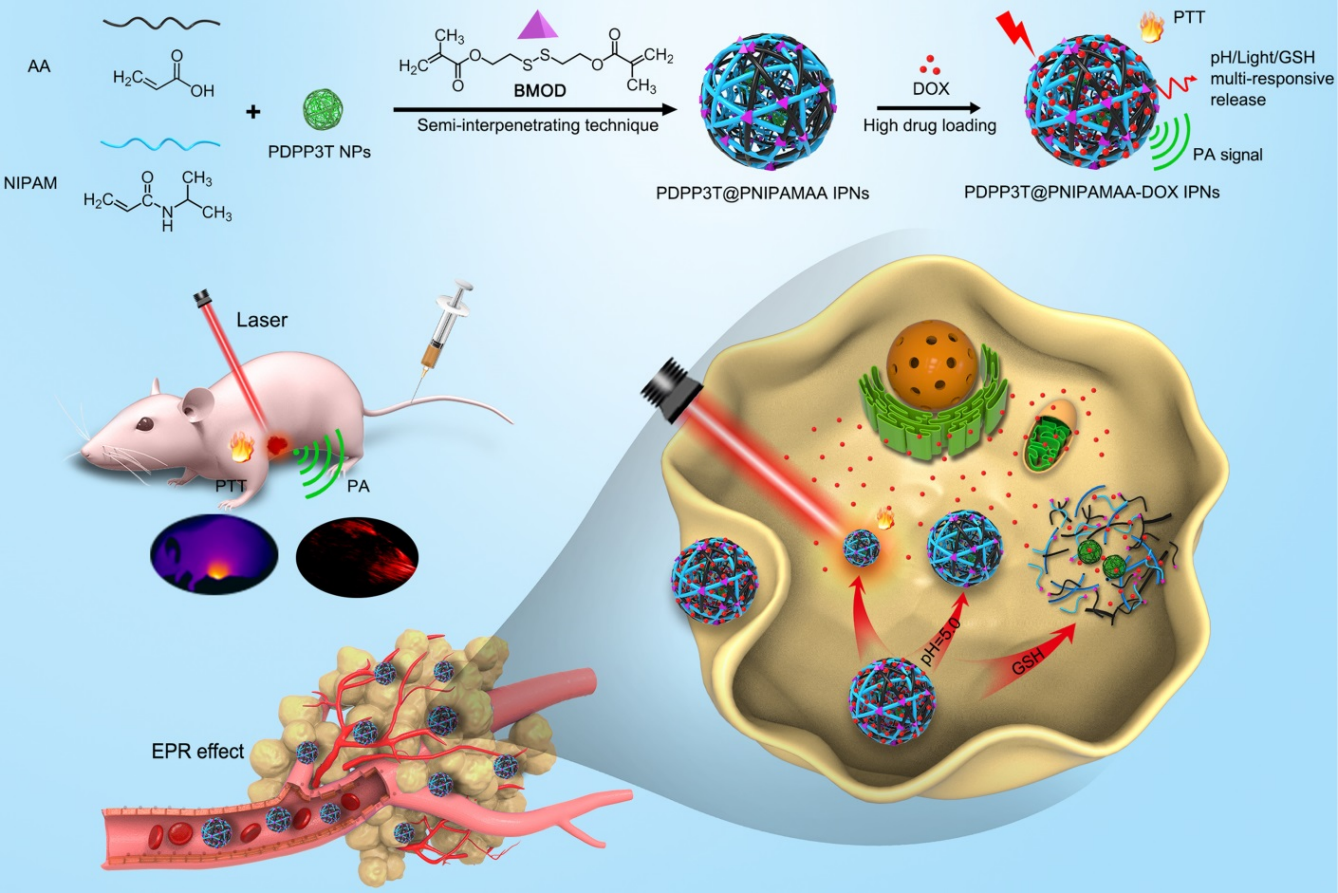
Schematic illustration to show the semi-interpenetrating synthesis of PDPP3T@PNIPAMAA-DOX IPNs and the PAI-guided chemo/photothermal combined therapy.

**Figure 2:**
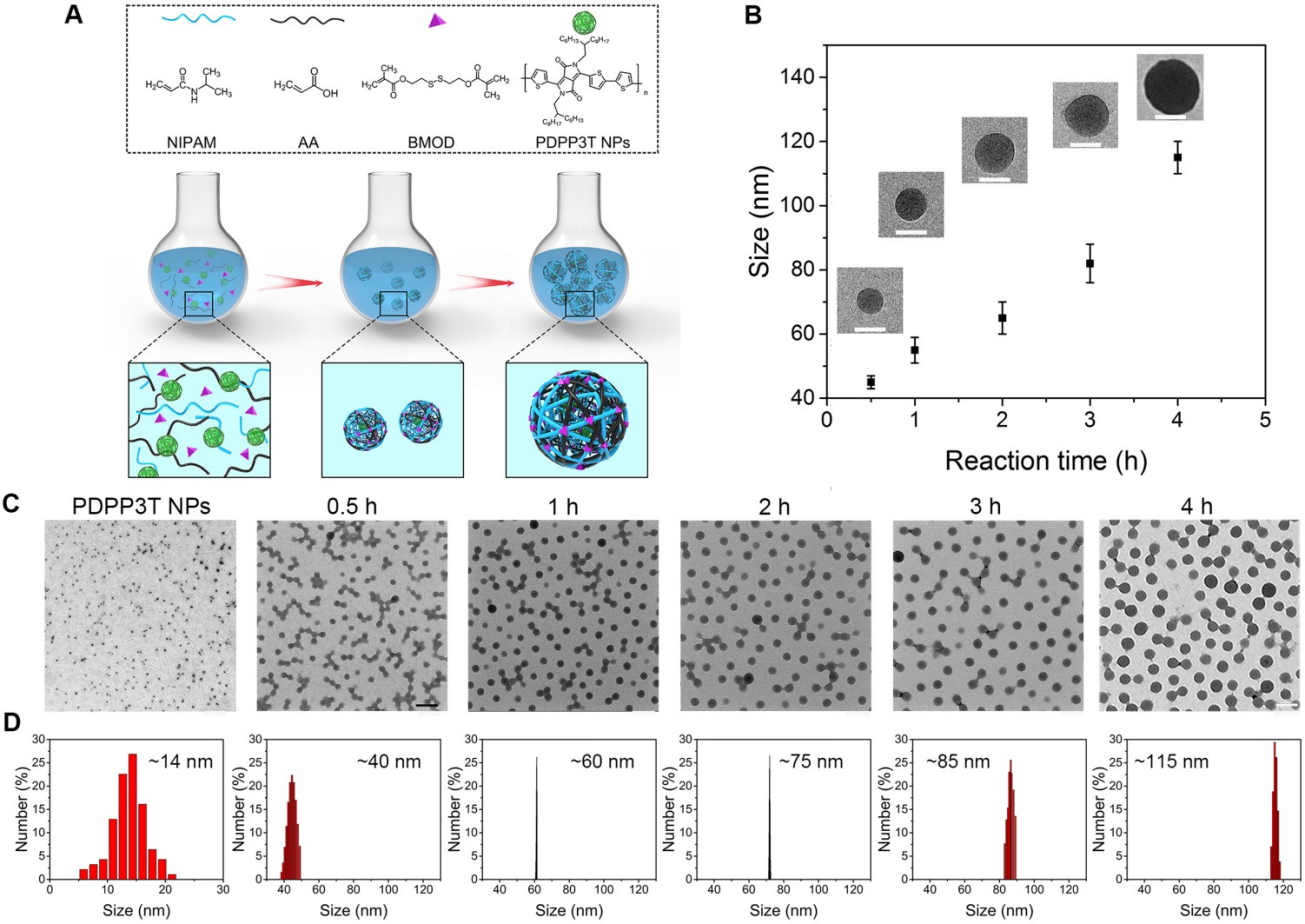
** Controllable preparation and growth mechanism of PDPP3T@PNIPAMAA IPNs. (A)** Schematic description for particle growth of PDPP3T@PNIPAMAA IPNs by semi-interpenetrating technique. **(B)** The size and photographs (inset) of PDPP3T@PNIPAMAA IPNs at different reaction times. Scale bar of all are 50 nm. **(C)** TEM images and **(D)** dynamic light scattering results of size-tunable PDPP3T@PNIPAMAA IPNs at different reaction times (0, 0.5, 1, 2, 3, 4 h). The scale bar of TEM images in Figure 2C are 200 nm.

Semi-interpenetrating polymer network (semi-IPN) is a technique that one polymer is crosslinked in the presence of another linear polymer to produce interlaced polymer networks and they cannot be separated unless the chemical structure are broken 24, 25. Up to now, the semi-IPN strategy has not been so far applied in the preparation of SPNs and no publication has appeared concerning it. Herein, to achieve excellent structural stability, better control of nanoparticle size and dispersity, high drug loading capacity, as well as multi-stimuli responsive chemo/ photothermal synergistic therapy, a facile *in-situ* interpenetrating strategy was proposed for the generation of semiconducting polymer nanoparticles (PDPP3T NPs) using *N*-isopropylacrylamide (NIPAM) and acrylic acid (AA) as monomer, bis(2-methacryloyl)oxyethyl disulfide (BMOD) as crosslinker (Figure 1 and Figure 2A) 26. The facile semi-penetrating strategy could form pH/light/GSH multi-responsive PDPP3T@PNIPAMAA IPNs, in which poly(NIPAM-*co*-AA) (PNIPAMAA) was crosslinked and interlaced with PDPP3T NPs chains to avoid the dissociation issues. The particle size of PDPP3T@PNIPAMAA IPNs could also be precisely controlled by adjusting reaction time. Most importantly, PDPP3T@PNIPAMAA IPNs possessed significantly high drug loading efficiency for cancer drug DOX (~92.64%) due to their semi-interpenetrating framework. The obtained PDPP3T@PNIPAMAA-DOX IPNs show quick responses to pH/light/GSH stimuli for controlled drug release. *In vitro* and *in vivo* experiments indicated their excellent photoacoustic imaging capacity and combined chemo/photothermal therapeutic effectiveness.

## Results and Discussion

### Controllable preparation and characterization of PDPP3T@PNIPAMAA IPNs

Owing to the excellent near-infrared (NIR) absorbing performance, semiconducting polymer poly(diketopyrrolopyrrole-terthiophene) (PDPP3T) was chosen as PTT agent and synthesized via stille polymerization according to previous reported method 27. To realize multi-stimuli responsive functionality, *N*-isopropylacrylamide (NIPAM) and acrylic acid (AA) were used as thermo-responsive and pH-sensitive unit respectively. Disulfide-crosslinker bis(2-methacryloyl)oxyethyl disulfide (BMOD) was incorporated here as redox-sensitive segment and also to provide stable and crosslinking functionalization of PDPP3T NPs. The disulfide-crosslinked PNIPAMAA were then partially interlaced with PDPP3T NPs chains by *in-situ* semi-interpenetrating technique. Because PNIPAMAA interpenetrate with PDPP3T without chemical bond, it should be expected that PDPP3T@PNIPAMAA IPNs could simultaneously retain photothermal capacity and pH/light/GSH-responsiveness. As shown in Figure 1, drug loading PDPP3T@PNIPAMAA IPNs (PDPP3T@PNIPAMAA-DOX IPNs) can thus serve as the excellent photothermal agents for the simultaneous PA imaging and multi-stimuli responsive drug delivery for *in vivo* tumor therapy.

It has been found that nanoparticle size has significant influence on tissue distribution, pharmacokinetics, tumor accumulation, and therapeutic efficiency 28-30. Although surface-functionalized SPNs with various therapeutic and imaging properties have been designed for different biomedical applications, there are only a few techniques for fabricating multi-functionalized SPNs were investigated. Additionally, limited success in the precise size control of surface-functionalized SPNs and subsequent fabrication of highly uniform surface-functionalized SPNs was achieved.

To realize the precise size control of multi-responsive SPNs, *in-situ* semi-interpenetrating method was proposed here to produce multi-responsive SPNs with uniform size. PDPP3T@PSNiAA NPs prepared by coprecipitation of PDPP3T and amphiphilic polymer polystyrene-*b*-poly(*N*-isopropylacrylamide-*co*-acrylic acid) (PSNiAA) were used as control 12. As shown in Figure S1, PDPP3T@PSNiAA NPs shows 23-102 nm size with a relatively wide size distribution (standard deviation, 14.03%) from TEM analysis. In the *in-situ* semi-interpenetrating method, the preparation of the multi-responsive PDPP3T@PNIPAMAA IPNs was started from preparation of PDPP3T NPs by reprecipitation method. The obtained PDPP3T NPs were later used as seeds (nucleation centers) and mixed with NIPAM solution containing AA, cross-linker BMOD and the ammonium persulphate (APS) initiator (Figure 2A). The size changes in the multi-responsive PDPP3T@PNIPAMAA IPNs induced by increasing the reaction time were measured by TEM images and dynamic light scattering (DLS) measurement. As shown in Figure 2B-D, the size of PDPP3T@PNIPAMAA IPNs could be readily controlled by adjusting the polymerization time. It is evident that the particle size of PDPP3T@PNIPAMAA IPNs increases from 40 to 108 nm based on TEM analysis and from ~43 nm to 115 nm according to DLS data with increase of reaction time. A plot of size diameter versus time is also shown in Figure 2B, and it can be seen that the particle size of PDPP3T@PNIPAMAA IPNs gradually increase as the reaction proceeds. Specifically, the relative standard deviation of the average particle size (from TEM images) decreased from 21.7% for PDPP3T NPs seed to 6.1% for PDPP3T@PNIPAMAA IPNs at the reaction time of 1 h. Furthermore, the uniform size of PDPP3T@PNIPAMAA IPNs was further confirmed by DLS, which shows polydispersity index (PDI) around 0.037 at the reaction time of 1 h. The UV-vis absorption spectrum of synthesized PDPP3T@PNIPAMAA IPNs was shown in Figure S2. The obtained PDPP3T@PNIPAMAA IPNs show a broad and strong absorbance ranged from 600 nm to 900 nm, which was mainly attributed to PDPP3T segments.

The possible mechanisms for the formation of PDPP3T@PNIPAMAA IPNs with narrow size distribution is shown in Figure 2A. In semi-interpenetrating technique, PDPP3T NPs can act as seeds and steric stabilizer for the polymerization reaction, directing the particle size and colloidal stability of the system. As a result, cross-linked PNIPAMAA polymer layer was incorporated into PDPP3T NPs to form semi-interpenetrating polymer network between PNIPAMAA chains and PDPP3T backbones. Thus well-defined PDPP3T@PNIPAMAA IPNs with monodispersed size distribution could be expected to form due to the widely appreciated feature of dispersion polymerization 31, 32. Furthermore, the particle loading number of PDPP3T NPs in PDPP3T@PNIPAMAA IPNs was calculated to be ~2 according to equation 1 in the experimental section.

### Nanoparticle stability of PDPP3T@PNIPAMAA IPNs

**Figure 3:**
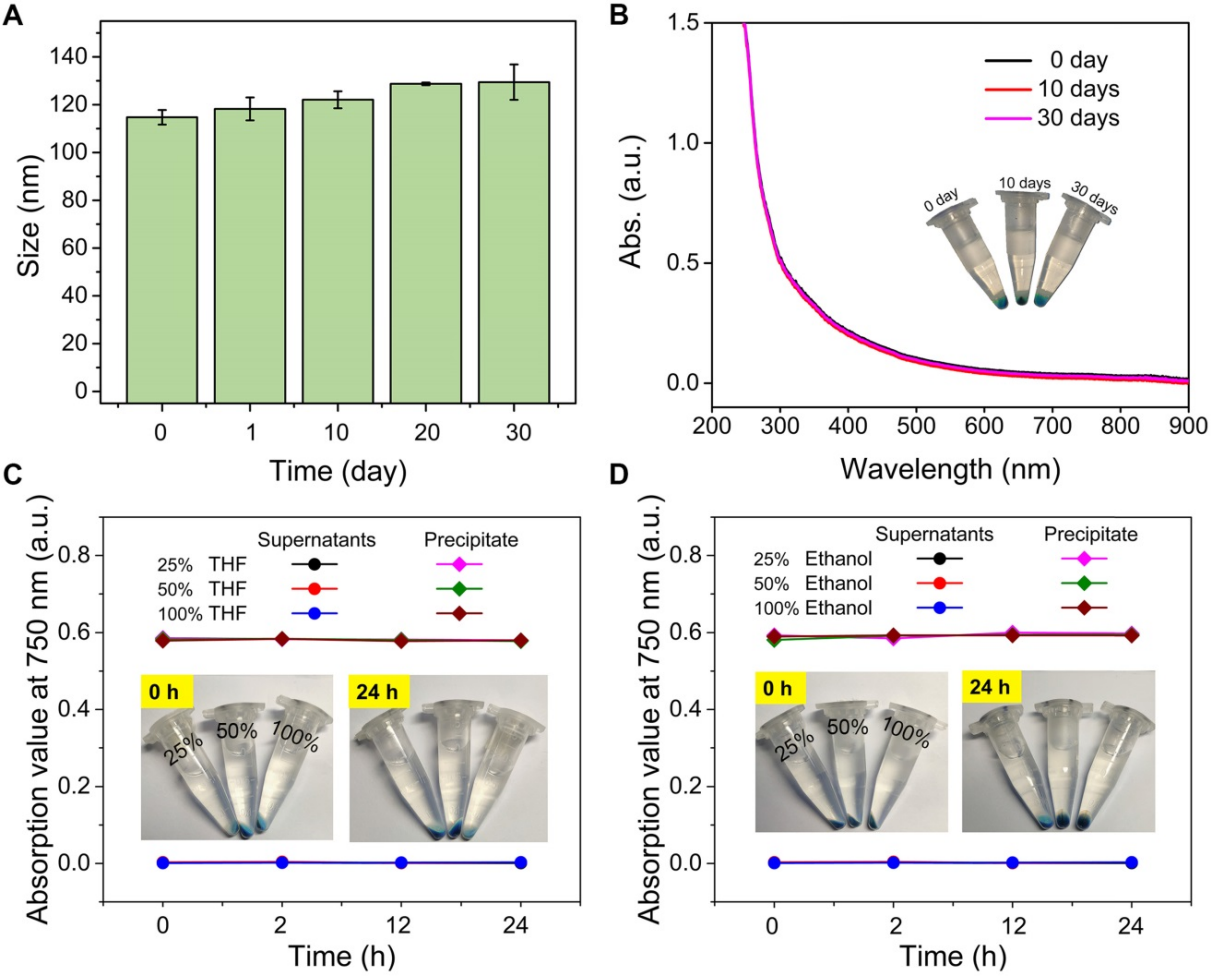
** Nanoparticle stability of PDPP3T@PNIPAMAA IPNs. (A)** The hydrodynamic diameter of PDPP3T@PNIPAMAA IPNs as a function of storage time. **(B)** The UV spectra of PDPP3T@PNIPAMAA IPNs supernatant and photographs (inset) of centrifugal PDPP3T@PNIPAMAA IPNs at different storage time (0, 10 and 30 days). The absorption of PDPP3T@PNIPAMAA IPNs supernatant and precipitate at 750 nm after incubated in aqueous solution with increasing content of **(C)** THF and **(D)** ethanol for different times. Insets: photographs of PDPP3T@PNIPAMAA IPNs incubated with different content of THF and ethanol for 24 h after centrifugation.

Since most functionalized SPNs were prepared by coprecipitation of semiconducting polymer and amphiphilic functional polymer through hydrophobic interactions, amphiphilic polymer molecules in the SPNs are susceptible to dissociate from SPNs due to relatively weak physical interactions. To overcome these issues, *in-situ* semi-interpenetrating polymerization of AA, NIPAM and BMOD in the presence of PDPP3T NPs was proposed to form PDPP3T@PNIPAMAA IPNs. The zeta potential of PDPP3T@PNIPAMAA IPNs was determined to ~-20 mV (Figure S3). We first test the colloidal stability of PDPP3T@PNIPAMAA IPNs in aqueous solution, PBS, Tris-HCl and DMEM by using DLS to monitor the size changes. As shown in Figure 3A and Figure S4, the absorption spectrum and the average particle size of PDPP3T@PNIPAMAA IPNs aqueous solution shows no obvious changes and no precipitation was observed during the investigation duration. PDPP3T@PNIPAMAA IPNs are also stable even in PBS, Tris-HCl, DMEM and FBS (Figure S5). The structural stability of PDPP3T@PNIPAMAA IPNs was further investigated by centrifugation method. Figure 3B showed that the supernatant of PDPP3T@PNIPAMAA IPNs after centrifugation at different storage time. Almost no characteristic UV absorbance of PDPP3T (750 nm) from the supernatant solution was observed after 30 days storage, which indicates that PNIPAMAA network and PDPP3T chains at least partially interpenetrates with each other at the molecular level and thus form highly stable and permanent structure. Specifically, PDPP3T@PNIPAMAA IPNs could be easily collected by centrifugation and be redissolved in aqueous solution directly (Figure S6), which is highly desirable for subsequent bioconjugation and could avoid tedious purification processes in most traditional preparation method for SPNs bioconjugates.

To further confirm the semi-interpenetrating structure of PDPP3T@PNIPAMAA IPNs, we incubated PDPP3T@PNIPAMAA IPNs in aqueous solution with increasing content of organic solvent such as ethanol and THF. As shown in Figure 3C-D, the UV spectra of PDPP3T@PNIPAMAA IPNs precipitate remain nearly the same after incubated with pure ethanol and THF for 24 h and the supernatant do not show any absorbance at 750 nm. On the basis of the above results, we propose that PDPP3T@PNIPAMAA IPNs undergoes a crosslinking polymerization process of PNIPAMAA and their simultaneous entangling with the linear PDPP3T chain in the nanoparticles to form the semi-interpenetrating structure. The final PDPP3T@PNIPAMAA IPNs have an interlocked network with “reinforced-concrete” structure, in which PNIPAMAA and PDPP3T form permanent semi-interpenetrating structures (physical cross-links) and prevent the occurrence of nanoparticles dissociation to a great extent. Additionally, the centrifuged nanoparticles from ethanol or THF can further be redispersed in water again.

### Photothermal property and PA imaging capacity of PDPP3T@PNIPAMAA IPNs

**Figure 4:**
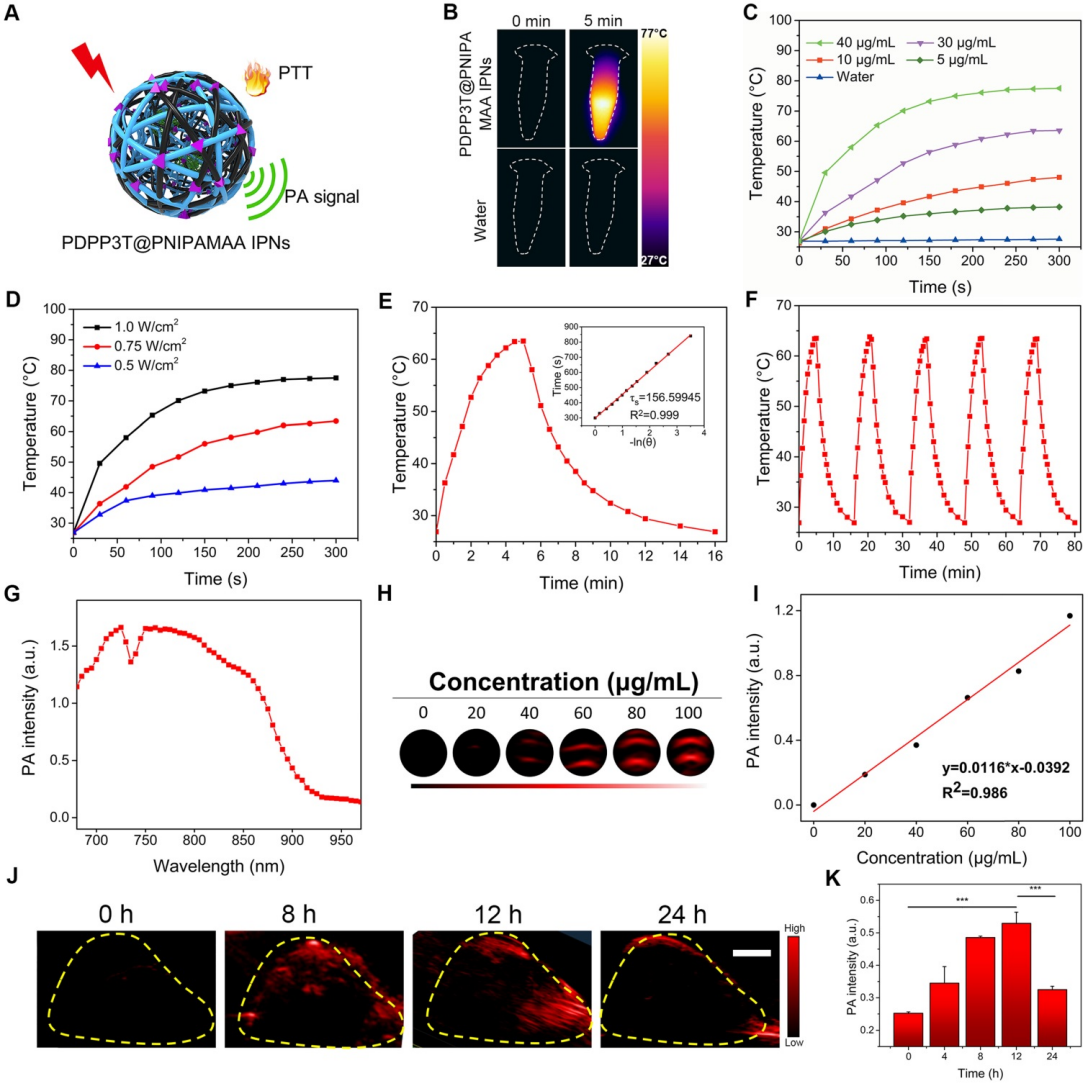
** Photothermal and PA properties of PDPP3T@PNIPAMAA IPNs. (A)** Schematic illustration of photothermal/photoacoustic property of PDPP3T@PNIPAMAA IPNs. **(B)** IR thermal images of PDPP3T@PNIPAMAA IPNs (40 μg/mL) and water with 808 nm laser irradiation. Temperature elevation of PDPP3T@PNIPAMAA IPNs **(C)** at various concentrations under 808 nm irradiation at 1 W/cm^2^ and **(D)** with different laser power densities at 40 μg/mL. **(E)** Photothermal effect of PDPP3T@PNIPAMAA IPNs with 808 nm laser irradiation, which was shut off after reaching the balanceable temperature. Inset: corresponding relationship between cooling time and negative natural logarithm of temperature driving force obtained from the cooling stage of Figure E. **(F)** Temperature elevation of PDPP3T@PNIPAMAA IPNs under 808 nm laser irradiation for five light on/off cycles. **(G)** PA spectrum of PDPP3T@PNIPAMAA IPNs. **(H)** The PA images of PDPP3T@PNIPAMAA IPNs with different concentrations in low-PA-density tubes. **(I)** The relationship of PA intensity and concentration of PDPP3T@PNIPAMAA IPNs. **(J)*** In vivo* PA images and intensity of PDPP3T@PNIPAMAA IPNs. **(K)*** In vivo* PA signal of PDPP3T@PNIPAMAA IPNs in tumor area as a function of post injection time (***P < 0.001). The scale bars of all images are 2 mm.

To study the photothermal conversion ability of PDPP3T@PNIPAMAA IPNs, the temperature elevation of PDPP3T@PNIPAMAA IPNs at different concentrations was recorded via IR camera (Figure 4A-C). PDPP3T@PNIPAMAA IPNs exhibited obvious temperature elevations under 5 min laser irradiation, while negligible temperature increase was detected for ultrapure water as control. Figure 4D demonstrates a laser power density-dependent temperature increase of the PDPP3T@PNIPAMAA IPNs, in which the temperature of PDPP3T@PNIPAMAA IPNs increased rapidly from 27 °C to 60 °C with a laser power density of 0.75 W/cm^2^. The photothermal conversion efficiency of PDPP3T@PNIPAMAA IPNs was further calculated to be 51.2% according to reported method (Figure 4E) 33. Additionally, the photothermal stability of PDPP3T@PNIPAMAA IPNs was investigated by applying five heating/cooling circles. As shown in Figure 4F, the temperature of PDPP3T@PNIPAMAA IPNs can increase to the same level after five circles of repeated irradiation. The excellent photothermal ability and good photothermal stability of PDPP3T@PNIPAMAA IPNs thus indicate their great potential as highly effective PTT agent.

The potential of PDPP3T@PNIPAMAA IPNs as PA imaging agent was tested in solution and in HeLa tumor *in vivo*. Figure 4G revealed that PDPP3T@PNIPAMAA IPNs exhibited strong PA signal in the broad NIR region from 680 nm to 850 nm, which was beneficial for *in vivo* imaging applications. The PA intensity of PDPP3T@PNIPAMAA IPNs upon 750 nm excitation at a series of concentrations (0, 20, 40, 60, 80, 100 μg/mL) were determined and a good linearity between the PA intensity and PDPP3T@PNIPAMAA IPNs concentrations was shown in Figure 4H-I. The *in vivo* PA imaging of PDPP3T@PNIPAMAA IPNs was further evaluated by systemic administration of PDPP3T@PNIPAMAA IPNs (200 μg/mL, 150 μL) into HeLa tumor-bearing mice. As shown in Figure 4J-K, the PA signal of PDPP3T@PNIPAMAA IPNs in tumor tissues gradually increased with time and reaches the maximum after 12 h injection. Examination of the *ex vivo* PA biodistribution verified that PDPP3T@PNIPAMAA IPNs could preferentially accumulated at the tumor site (Figure S7), which is assumed to be mediated by an enhanced permeability and retention effect 30, 34 and could reduce the side effect caused by hyperthemia.

### Near-infrared light/pH-triggered release of DOX from PDPP3T@PNIPAMAA-DOX IPNs

The physical and chemical combination methods with combined excellent properties such as various sensitivity and photothermal capacity to meet specific needs have shown great promising for theranositic applications. Semi-interpenetrating structure containing interlaced multipolymers not only have advantages in offering enhanced mechanical strength to resist phase separation compared to homopolymer network 35, 36, it is also reported that hydrophilic three-dimensional interpenetrating structure are capable of absorb a large quantity of water or biological drugs 37, 38. Herein, DOX was chosen as the model drug and loaded into PDPP3T@PNIPAMAA IPNs. It was found that the encapsulation efficiency of PDPP3T@PNIPAMAA IPNs was significantly increased (~92.64%) compared to PDPP3T@PSNiAA NPs (~24.1%) under the same loading conditions. Such a high drug loading capacity can be attributed to the excellent loading capacity of PDPP3T@PNIPAMAA IPNs, which could provide adequate association positions for drug binding. Furthermore, the cross-linked network structure of PDPP3T@PNIPAMAA IPNs can also promote DOX encapsulation through physical entrapment of DOX inside PDPP3T@PNIPAMAA IPNs matrix and finally enable a high DOX drug loading content.

**Figure 5:**
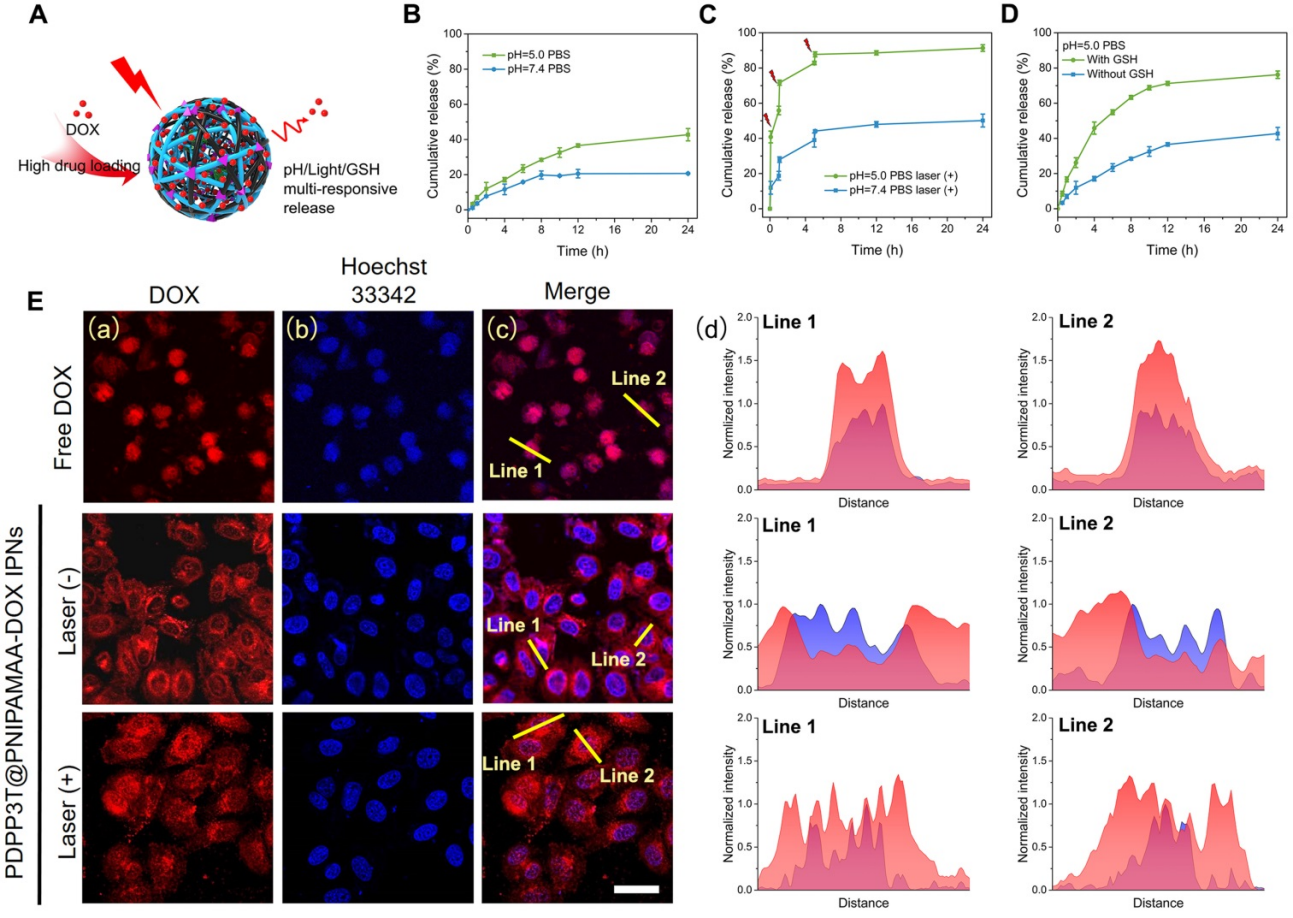
** Drug release behavior of PDPP3T@PNIPAMAA-DOX IPNs. (A)** Schematic illustration of drug release behavior of PDPP3T@PNIPAMAA-DOX IPNs. DOX release profiles of PDPP3T@PNIPAMAA-DOX IPNs **(B)** without and **(C)** with 808 nm laser irradiation in different pH values of PBS buffer respectively. **(D)** GSH-triggered DOX release of PDPP3T@PNIPAMAA-DOX IPNs at PBS (pH 5.0). **(E)** Representative CLSM images of intracellular NIR laser-triggered drug release from PDPP3T@PNIPAMAA-DOX IPNs. (a) Red channel for DOX, (b) blue channel for Hoechst. (c) Overlay fluorescence image of (a) and (b). (d) Fluorescence intensity profile of regions of interest (yellow line in c) across the lines. The scale bars are all 40 µm.

The drug release behavior of DOX loaded pH/light/GSH responsive PDPP3T@PNIPAMAA IPNs (PDPP3T@PNIPAMAA-DOX IPNs) are studied at pH 5.0 and 7.4, respectively (Figure 5A). As shown in Figure 5B, PDPP3T@PNIPAMAA-DOX IPNs exhibited slow DOX release (~ 20%) at pH 7.4, while ~40% of DOX was released from PDPP3T@PNIPAMAA-DOX IPNs at pH 5.0. The pH-responsive release properties of PDPP3T@PNIPAMAA-DOX IPNs is probably due to the decreased interaction between DOX and PNIPAMAA and the increased solubility of DOX in acidic medium 39, 40. It can be found that DOX release was significantly promoted and reached ~90% under laser irradiation at pH 5.0 (Figure 5C), which could be attributed to the photothermal capacity of PDPP3T@PNIPAMAA-DOX IPNs and the thermo-sensitivity of PNIPAMAA. Specifically, the NIR light-triggered DOX release at acidic medium (pH 5.0) seemed is much more rapid than that at neutral condition (pH 7.4), which was beneficial for the controlled drug release in tumor tissues 41. Considering that the GSH concentration in many human cancers are significantly higher than that in normal cells, the DOX release of PDPP3T@PNIPAMAA-DOX IPNs under 10 mM GSH condition was shown in Figure 5D. It could be observed that PDPP3T@PNIPAMAA-DOX IPNs showed a much more rapid DOX release with addition of 10 mM GSH, which demonstrated that the release of DOX from PDPP3T@PNIPAMAA-DOX IPNs could be triggered by GSH due to the disulfide linkages in BMOD crosslinker. These results highlight that PDPP3T@PNIPAMAA-DOX IPNs have a great potential as excellent multi-responsive drug nanocarriers.

The intracellular NIR light-triggered DOX release of PDPP3T@PNIPAMAA-DOX IPNs were investigated in HeLa cells using CLSM. As shown in Figure 5E, free DOX penetrate HeLa cells and then cumulated in the nuclei quickly. Compared to free DOX as control, PDPP3T@PNIPAMAA-DOX IPNs without laser irradiation are more concentrated in cytoplasm and a mild fluorescence from DOX was observed in nuclei in HeLa cells. NIR light triggered DOX release in HeLa cells was shown in Figure 5E. The significantly enhanced fluorescence from DOX in the cell nuclei can be detected when HeLa cells were exposed to NIR light irradiation, which should be due to the thermal sensitivity of PNIPAMAA network and subsequent drug diffusion into the nuclei. The results show that laser irradiation can triggered the controllable release of DOX from PDPP3T@PNIPAMAA-DOX IPNs into the nucleus rather than cumulated in nucleus directly to kill cancer cells, which indicate that PDPP3T@PNIPAMAA IPNs can serve as stimuli-responsive drug carrier and reduce the side effect of DOX.

### *In vitro* cytotoxicity of PDPP3T@PNIPAMAA-DOX IPNs

**Figure 6:**
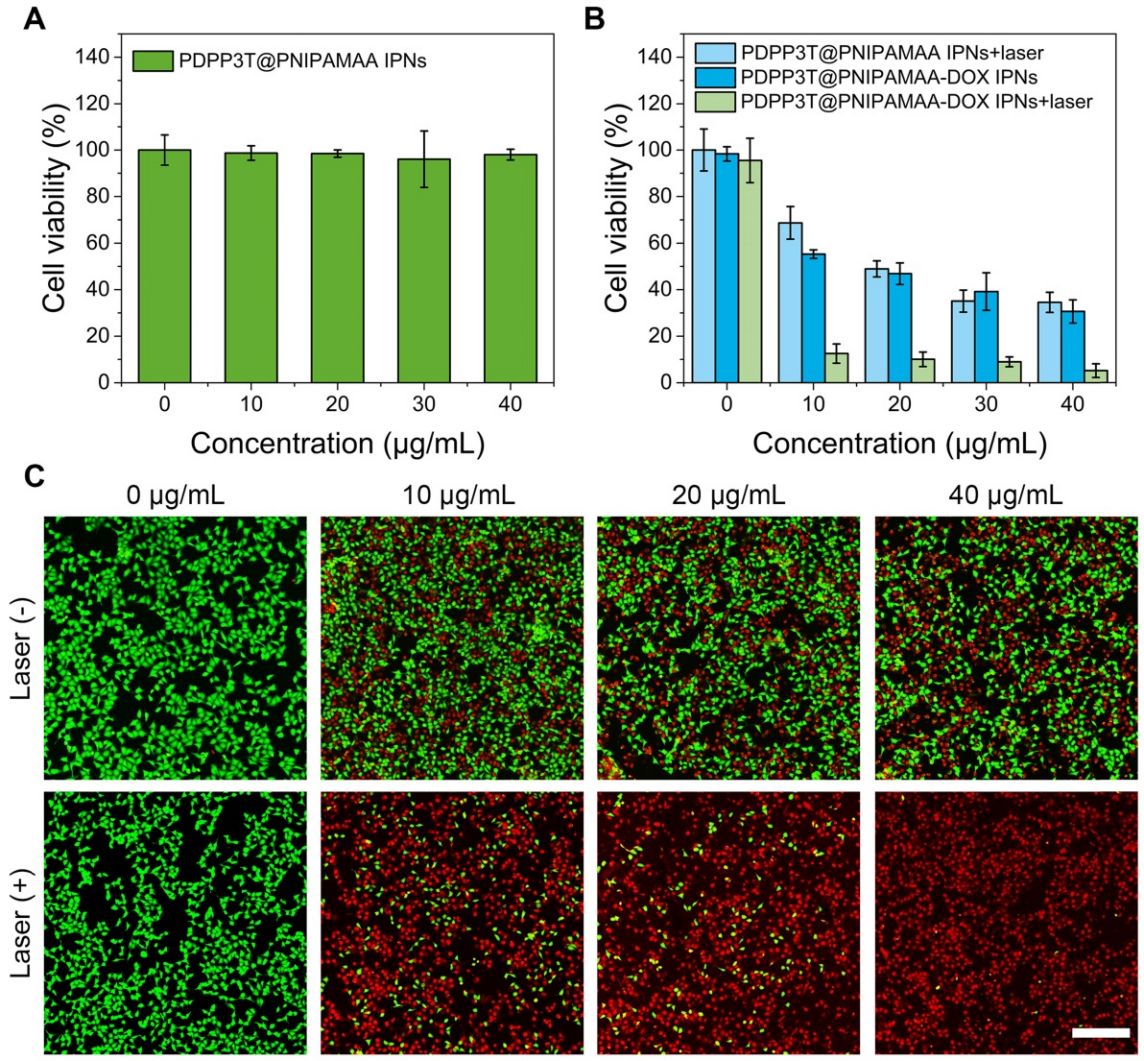
***In vitro* chemo/photothermal combined therapy of PDPP3T@PNIPAMAA-DOX IPNs. (A)** Viabilities of HeLa cells incubated with PDPP3T@PNIPAMAA IPNs. **(B)** Viabilities of HeLa cells treated with PDPP3T@PNIPAMAA IPNs and PDPP3T@PNIPAMAA-DOX IPNs with or without 808 nm laser irradiation. **(C)** CLSM images of HeLa cells stained with calcein-AM/PI to visualize cell viabilities treated by PDPP3T@PNIPAMAA-DOX IPNs with or without laser irradiation. The scale bars are all 200 µm.

To evaluate PDPP3T@PNIPAMAA IPNs as photothermal agent and effective drug carrier for theranostic applications, the cytotoxicity against HeLa cells were investigated by the standard methylthiazolyltetrazolium (MTT) assay. As shown in Figure 6A, PDPP3T@PNIPAMAA IPNs exhibited no cytotoxicity toward HeLa cells, which indicated their good biocompatibility. To determine the chemo/photothermal therapy effect, PDPP3T@PNIPAMAA-DOX IPNs were incubated with HeLa cells with or without NIR light irradiation (Figure 6B). As expected, PDPP3T@PNIPAMAA IPNs could inhibit the growth of HeLa cells under NIR laser irradiation at PDPP3T@PNIPAMAA IPNs concentration of 20 μg/mL (∼50% of cell viability). Free DOX exhibited significant cytotoxic effect upon HeLa cells (Figure S8), while PDPP3T@PNIPAMAA-DOX IPNs showed much lower cytotoxicity to cancer cells in the dark with equivalent dose of DOX. Remarkably, PDPP3T@PNIPAMAA-DOX IPNs upon irradiation showed significantly killing efficacy to HeLa cells (less than 10% of cell viability) compared to single chemotherapy or photothermal therapy group. The enhanced HeLa cell ablating ability by combined chemo/photothermal therapy of PDPP3T@PNIPAMAA-DOX IPNs was verified by CLSM using calcein-AM and propidium iodide (PI) staining. Figure 6C shows that HeLa cells incubated with PDPP3T@PNIPAMAA-DOX IPNs without laser irradiation exhibited moderately reduced viability. In contrast, markedly reduced cells viability was observed after PDPP3T@PNIPAMAA-DOX IPNs+laser treatment of HeLa cells even at a low concentration of PDPP3T@PNIPAMAA-DOX IPNs (10 μg/mL).

### *In vivo* antitumor activity of PDPP3T@PNIPAMAA-DOX IPNs

**Figure 7:**
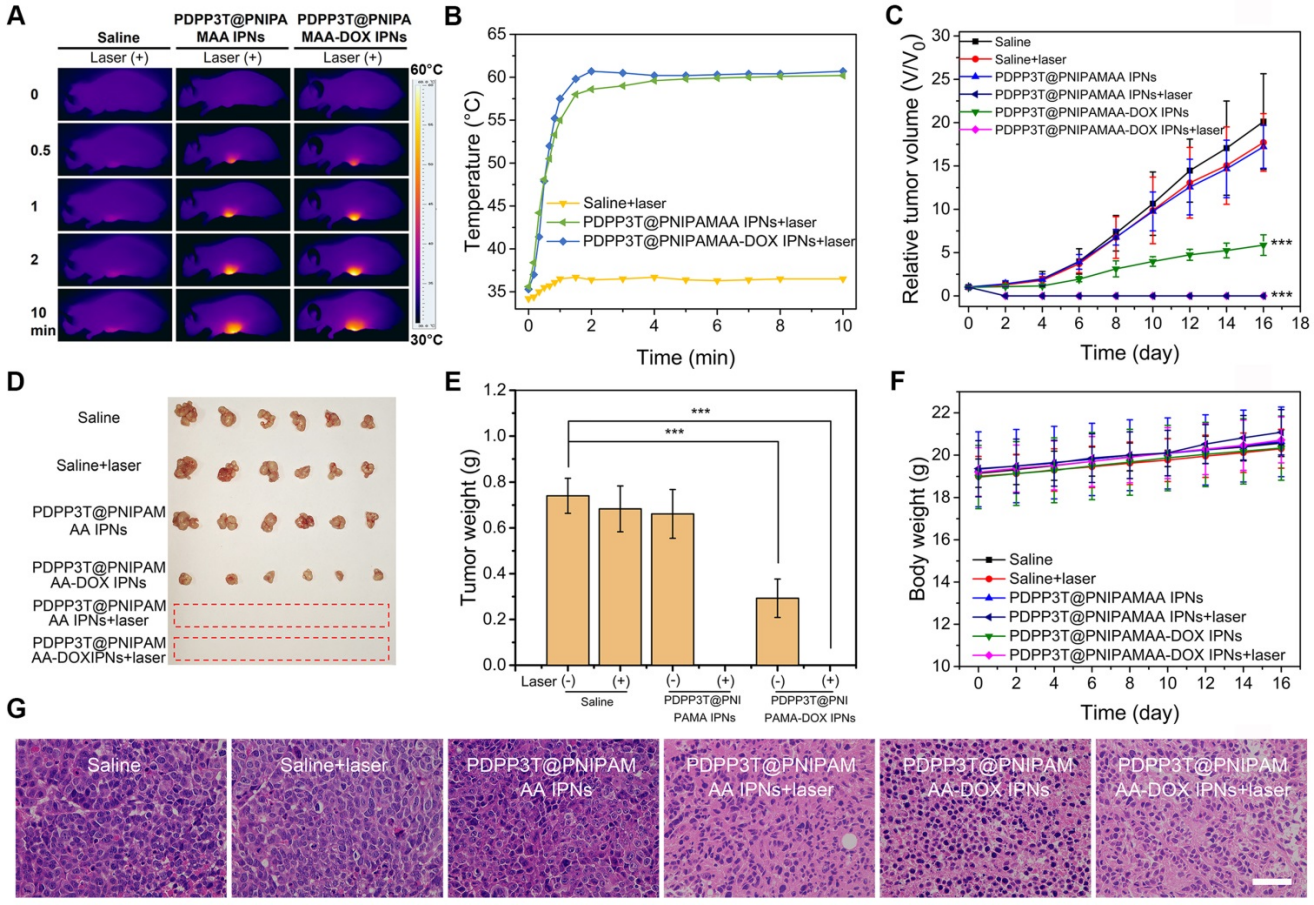
***In vivo* photothermal performance and combined chemo/photothermal therapy efficacy of PDPP3T@PNIPAMAA-DOX IPNs. (A)** IR thermal images of HeLa tumor-bearing mice after injection of saline, PDPP3T@PNIPAMAA IPNs, and PDPP3T@PNIPAMAA-DOX IPNs (DOX injection dose at ~20 μg) with NIR laser irradiation. **(B)** The temperature elevation at the tumor site of HeLa tumor-bearing mice after intravenously injection of saline, PDPP3T@PNIPAMAA IPNs, and PDPP3T@PNIPAMAA-DOX IPNs with NIR laser irradiation. **(C)** Tumor growth curves (***P < 0.001) and **(D)** tumor photographs after different treatments indicated. **(E)** Tumor weight after different treatments. (***P < 0.001). **(F)** Body weight data of tumor-bearing mice after various treatments indicated. **(G)** The H&E images of tumor slices of mice treated with saline, PDPP3T@PNIPAMAA IPNs, and PDPP3T@PNIPAMAA-DOX IPNs at 24 h after irradiation (808 nm, 1 W/cm^2^, 10 min). The scale bars are all 100 µm.

Encouraged by the excellent structure stability and remarkably enhanced cell-killing ability, we next investigated the *in vivo* combined chemo/ photothermal therapeutic efficacy of PDPP3T@PNIPAMAA-DOX IPNs. HeLa tumor-bearing nude mice were used as model animals. Figure 7A-B revealed that the tumor temperature of PDPP3T@PNIPAMAA-DOX IPNs-treated mice rapidly reached 60 °C within 2 min, which confirmed the excellent photothermal property of PDPP3T@PNIPAMAA-DOX IPNs. The *in vivo* antitumor efficacy of PDPP3T@PNIPAMAA-DOX IPNs for combined chemo/photothermal treatments were further studied. Tumor growth was significant for control groups receiving saline with laser irradiation, or blank PDPP3T@PNIPAMAA IPNs and saline without laser irradiation (Figure 7C-E). While tumors of the mice intravenously injected with PDPP3T@PNIPAMAA-DOX IPNs but no laser irradiation was partially inhibited. Notably, tumors of the mice treated with PDPP3T@PNIPAMAA-DOX IPNs plus laser irradiation are totally ablated with one treatment and the tumors showed no recurrence over the duration of experimental period. The tumor growth was also significantly suppressed by PDPP3T@PNIPAMAA IPNs with laser irradiation due to the excellent photothermal property of PDPP3T@PNIPAMAA IPNs. These results revealed that PDPP3T@PNIPAMAA-DOX IPNs can serve as chemo/photothermal agent for highly efficient cancer therapy. Moreover, the body weights of mice models showed slight growth for all the groups (Figure 7F), demonstrating good biocompatibility of PDPP3T@PNIPAMAA IPNs. The hematoxylin and eosin (H&E) stained assay indicated that negligible tumor cell apoptosis was detected in mice treated with saline with or without irradiation, and blank PDPP3T@PNIPAMAA IPNs with no irradiation (Figure 7G). Significantly, substantial tumor cells necrosis was found with condensed and fragmented cell nuclei for combined chemo/photothermal therapy group, while PDPP3T@PNIPAMAA-DOX IPNs without irradiation only caused partial apoptosis of tumor cells. In addition, the H&E staining analyses also revealed that PDPP3T@PNIPAMAA-DOX IPNs treatment induced no obvious toxic side effects to major organs (Figure S9).

## Conclusion

A semi-interpenetrating strategy to prepare highly stable and multi-responsive PDPP3T@PNIPAMAA IPNs is first demonstrated. This approach is simple, easily operated and reproducible. The particle sizes are controllable and their polydispersity index is extremely low. A permanent entangling network with “reinforced-concrete” structures for PDPP3T@PNIPAMAA IPNs has been simultaneously formed, which could avoid particle dissociation and is resistant to organic solvent such as ethanol and THF. Due to the presence of three-dimensional cross-linked structure, PDPP3T@PNIPAMAA IPNs show significantly high drug loading efficiency. A combination of pH/light/GSH-responsiveness, excellent photothermal efficiency and bright PA imaging capacity was obtained for PDPP3T@PNIPAMAA IPNs since PDPP3T and PNIPAMAA entangled together with no chemical bond and both constituents may retain their own properties. *In vitro* and *in vivo* results indicate that PDPP3T@PNIPAMAA-DOX IPNs are able to release drugs at controlled rate by pH/light/GSH regulation and offer PAI-guided chemo/photothermal combined therapy with excellent therapeutic efficacy. This approach may be generally extended for the preparation of a wide range of organic polymer nanoparticles to achieve ultrahigh structural stability, precise particle size controllability and excellent drug loading capacity.

## Materials and Methods

### Materials

Acrylic acid (AA), *N*-isopropylacrylamide (NiPAM), ammonium persulphate (APS), bis(2-methacryloyl)oxyethyl disulfide (BMOD) and sodium dodecyl sulfate (SDS) were purchased from Sigma-Aldrich (St. Louis, USA). All chemicals in cell experiments were purchased from Keygen Biotech Co., Ltd (Nanjing, China).

### Characterization

The UV-vis absorption spectra were examined on a UV-3600 spectrophotometer (Shimadzu, Japan). Hitach HT7700 transmission electron microscope was used to collecte the transmission electron microscopy (TEM) images. The diameters of nanoparticles were tested by a Brookhaven Zeta PALS. The confocal laser scanning microscope (CLSM) images were obtained from an FV1000 scanning microscope (Olympus, Japan). The PA images and intensity were obtained from a photoacoustic/ultrasonic imaging system (Vevo LAZR). The Fotric 225 photothermal camera was used to record temperature and thermal images.

### Preparation of PDPP3T NPs

In a representative preparation, 0.1 mg PDPP3T was dissolved in 6 mL anhydrous THF. Then, it was quickly injected into ultrapure water upon ultrasonic conditions. THF was removed by vacuum rotary evaporator and PDPP3T NPs aqueous solution was prepared by filtration through membrane filter.

### Preparation of PDPP3T@PNIPAMAA IPNs

Typically, *N*-isopropylacrylamide (NIPAM) (100 mM, 6 mL), acrylic acids (100 mM, 0.67 mL), bis(2-methacryloyl)oxyethyl disulfide (50 mM, 1.2 mL), sodium dodecyl sulfate (SDS) (100 mM, 100 μL) aqueous solution and 2 mL of PDPP3T NPs (30 μg/mL) was added to a flask. After nitrogen blowing for 20 min, ammonium persulphate (APS) (10 mM, 1 mL) was quickly injected into the mixture. The polymerization was reacted for 4 h at 72 °C. Finally, PDPP3T@PNIPAMAA IPNs with particle size of ~115 nm was collected via centrifugation (14000 rpm, 30 min) and purified with ultrapure water for three times to remove small molecules and bare PDPP3T NPs. Such PDPP3T@PNIPAMAA IPNs of this size were then used in the following *in vitro* and *in vivo* experiments unless otherwise stated.

### Calculation of PDPP3T NPs numbers in PDPP3T@PNIPAMAA IPNs

The total number of PDPP3T@PNIPAMAA IPNs and PDPP3T NPs were calculated according to equation (1) 42-44.


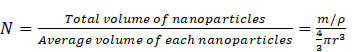
 (1)

Where *m* represents the total mass of nanopaticles, *ρ* is the particle density (~1 g/mL), *r* is the radius of single nanoparticle determined from TEM images.

In 2 mL PDPP3T@PNIPAMAA IPNs aqueous solution, the total number of PDPP3T NPs (*N_1_*) was calculated as following:


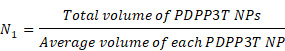



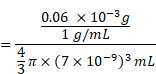


The total number of PDPP3T@PNIPAMAA IPNs (*N_2_*) was calculated as following:


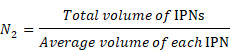



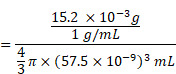


The ratio of PDPP3T NPs and PDPP3T@PNIPAMAA IPNs (*N_1_*/*N_2_*) is:


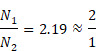


### Stability measurement of PDPP3T@PNIPAMAA IPNs

The supernatant of PDPP3T@PNIPAMAA IPNs dispersion system was taken after centrifugation at set intervals. The stability of PDPP3T@PNIPAMAA IPNs was investigated by monitoring the dispersibility and changes of the absorbance spectrum of the supernatant in ethanol and THF.

### Encapsulation and release of DOX from PDPP3T@PNIPAMAA-DOX IPNs

0.3 mg DOX and prepared PDPP3T@PNIPAMAA IPNs were mixed in 10 mL ultrapure water and stirred overnight at room temperature. DOX-loaded PDPP3T@PNIPAMAA IPNs (PDPP3T@PNIPAMAA-DOX IPNs) were collected by centrifugation (14000 rpm, 30 min) until there was no absorption of DOX in the supernatant.

LE was calculated as following:


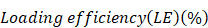
=


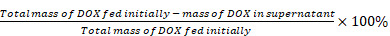
 (2)

The release behavior of DOX from PDPP3T@PNIPAMAA-DOX IPNs was studied by dialysis in PBS (pH = 7.4, 5.0). After 808 nm laser irradiation for 5 min (0.75 W/cm^2^), 0.5 mL dialysate was taken out at regular intervals and the released medium was replenished with equivolumetric PBS. Moreover, the PBS (pH = 5.0) with 10 mM GSH was applied to investigate the effect of GSH on drug release and the PBS (pH = 7.4) was used as control group.

The cumulative release ratio of drug was calculated as following equation:


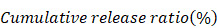
=


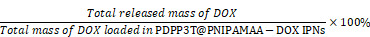


 (3)

### Confocal fluorescence imaging

HeLa cells were maintained with 5% CO_2_ for 24 h at 37 °C incubator. After removing the old medium, PDPP3T@PNIPAMAA-DOX IPNs was dispersed in 1 mL fresh medium and incubated with cells for another 6 h. The confocal laser scanning microscopy (CLSM) images were obtained via blue fluorescing (λ_ex_ = 405 nm) for Hoechst 33342 and red fluorescing (λ_ex_ = 488 nm) for DOX before and after 808 nm laser irradiation (0.75 W/cm^2^, 5 min). The cells cultured with free DOX was choose as the control group.

### Photothermal property of the PDPP3T@PNIPAMAA IPNs

PDPP3T@PNIPAMAA IPNs aqueous solution with various concentrations (5, 10, 30, 40 μg/mL) were respectively exposed with 808 nm at 1 W/cm^2^ for 5 min. The temperature changes of PDPP3T@PNIPAMAA IPNs (40 μg/mL) irradiated at different power densities (0.5, 0.75 and 1 W/cm^2^) were also recorded. The thermal stability of PDPP3T@PNIPAMAA IPNs was also verified through five heat-cooling cycles.

### *In vitro* cytotoxicity

HeLa cells plated in a 96-well plates with a density of 5×10^3^ cells per well were incubated with various concentrations of PDPP3T@PNIPAMAA IPNs and PDPP3T@PNIPAMAA-DOX IPNs for 24 h in dark. Subsequently, the cells were irradiated with 808 nm laser for 5 min (1 W/cm^2^). 50 μL of MTT reagent was added and the cells were incubated for another 4 h. The supernatant was moved and 200 μL DMSO added. The cell viabilities were measured using Bio Tek microplate reader.

### Animal model

All female nude mice (BALB/c) in experiments were purchased from OG Pharmaceutical. Co. Ltd (Nanjing, China). All experiments *in vivo* were performed in compliance with the guideline established and carried out according to the protocols approved by OG Pharmaceutical. Tumors were grown for ~21 days before applied for experiments.

### Photoacoustic property *in vitro* and *in vivo*

The samples containing different concentration (0, 20, 40, 60, 80, 100 μg/mL) of PDPP3T@PNIPAMAA IPNs were inflooded into low-PA-density polyethylene tubes respectively to record the intensity and images of PA signal. The working laser excitation wavelength was 750 nm.

For *in vivo* tumor PA imaging, tumor-bearing mice were injected with the PDPP3T@PNIPAMAA IPNs (200 μg/mL, 150 μL) through tail vein and the PA intensity and images were acquired under 750 nm laser irradiation at 0, 4, 8, 12 and 24 h.

### *In vivo* chemo/photothermal therapy

HeLa tumor-bearing nude mice (n = 6) were divided into six groups and then respectively injected via the tail vein with 150 μL of saline, PDPP3T@PNIPAMAA IPNs (200 μg/mL) and PDPP3T@PNIPAMAA-DOX IPNs (200 μg/mL). 12 hours later, the tumor site of corresponding groups were exposed to 808 nm laser for 10 min (1 W/cm^2^). The tumor slices of mice treated with saline, PDPP3T@PNIPAMAA IPNs, and PDPP3T@PNIPAMAA-DOX IPNs at 24 h after irradiation were stained with hematoxylin and eosin, moreover, the H&E images of major organs were obtained after 16 days of treatment.

### Statistical analysis

Results are presented as mean ± standard deviation. Statistical significance was determined by ANOVA and two-sample Student's *t*-test: *P* value <0.05 was considered significant.

## Supplementary Material

Supplementary figures and tables.
