# Supplementary material for: Highly stable semiconducting polymer nanoparticles for multi-responsive chemo/photothermal combined cancer therapy
Source: Theranostics. 2020 May 1;10(13):5966–78. doi: 10.7150/thno.43090 (PMC7254994; doi:10.7150/thno.43090)
Supplement: Supplementary file 1 — Supplementary figures and tables. [file thnov10p5966s1.pdf]

Supporting Information

Highly Stable Semiconducting Polymer Nanoparticles for Multi-Responsive Chemo/Photothermal Combined Cancer Therapy

Yu Xu[1], Xue Zhai[1], Peng Su[1], Tianqi Liu[1], Luyao Zhou[1], Jingjing Zhang[1], Biqing Bao[1]* and Lianhui Wang[1]*

1. Key Laboratory for Organic Electronics and Information Displays & Jiangsu Key Laboratory for Biosensors, Institute of Advanced Materials (IAM), Jiangsu National Synergetic Innovation Center for Advanced Materials (SICAM), Nanjing University of Posts & Telecommunications, Nanjing 210023, China

*Corresponding authors: iambqbao@njupt.edu.cn, iamlhwang@njupt.edu.cn

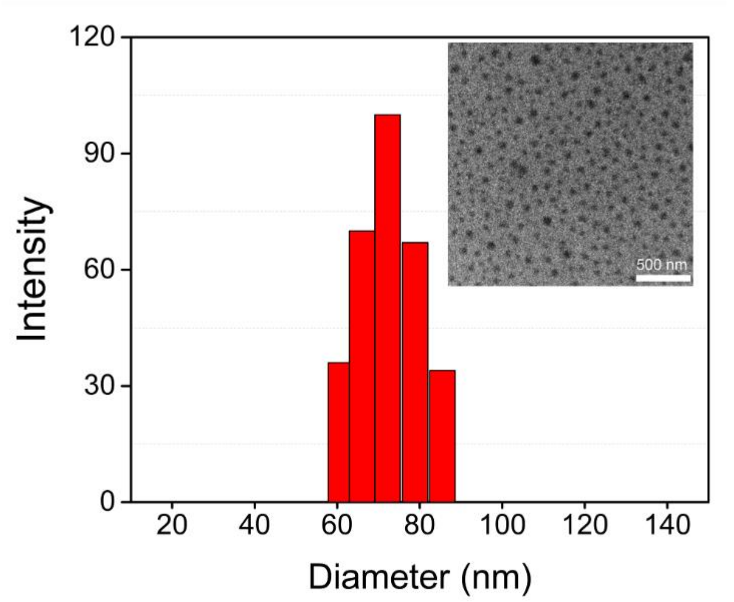

Figure S1. DLS data and TEM image (inset) of PDPP3T@PSNiAA NPs.

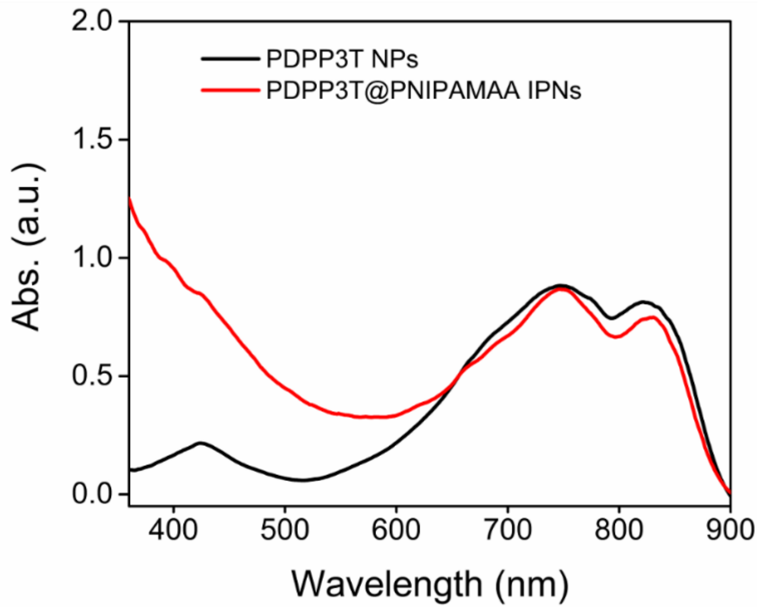

Figure S2. The UV spectra of PDPP3T NPs and PDPP3T@PNIPAMAA IPNs.

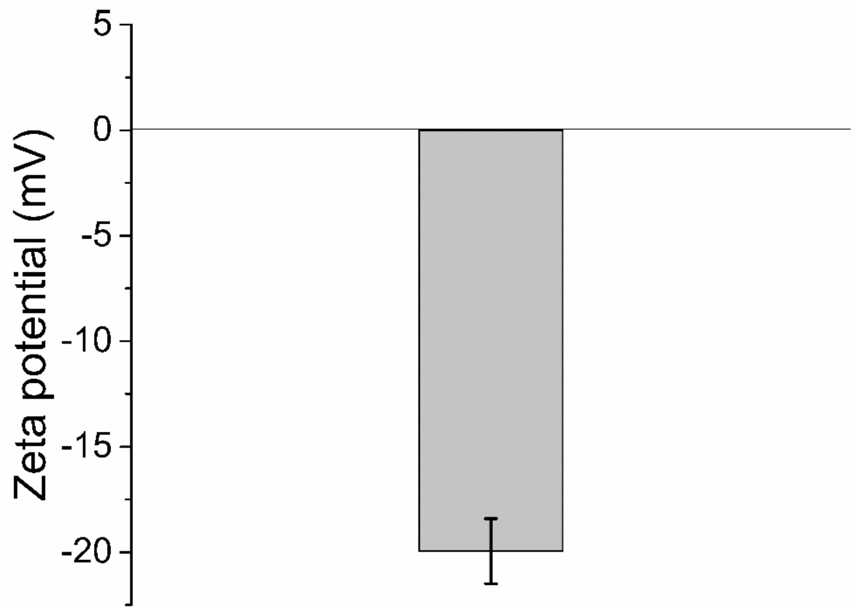

Figure S3. The zeta potential of PDPP3T@PNIPAMAA IPNs.

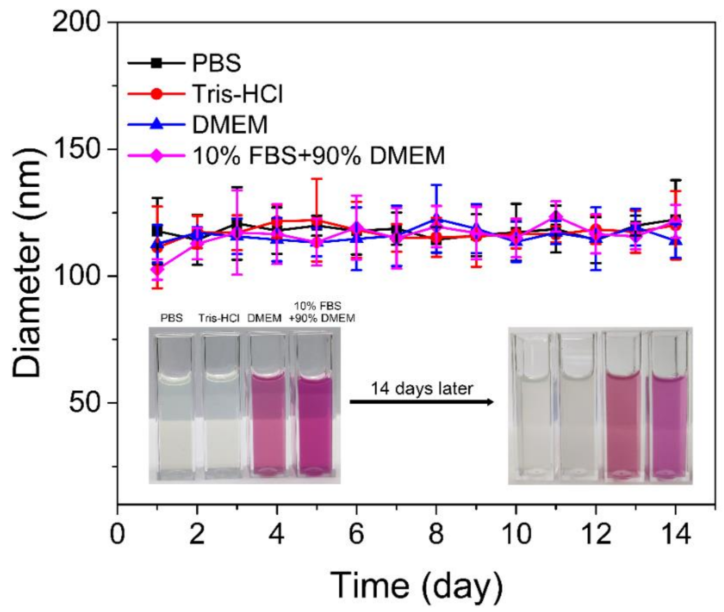

Figure S4. The photograph and DLS data of PDPP3T@PNIPAMAA IPNs in PBS, Tris-HCl, DMEM and 10% FBS+90% DMEM for 14 days.

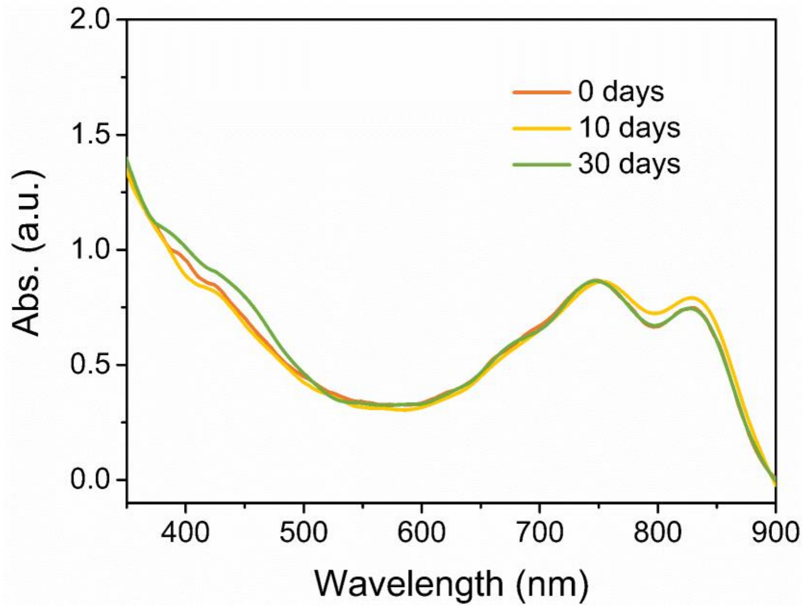

Figure S5. The UV spectra of PDPP3T@PNIPAMAA IPNs for 30 days.

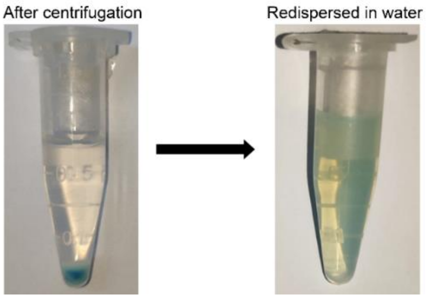

Figure S6. The photograph of PDPP3T@PNIPAMAA IPNs redispersed in aqueous solution after centrifugation.

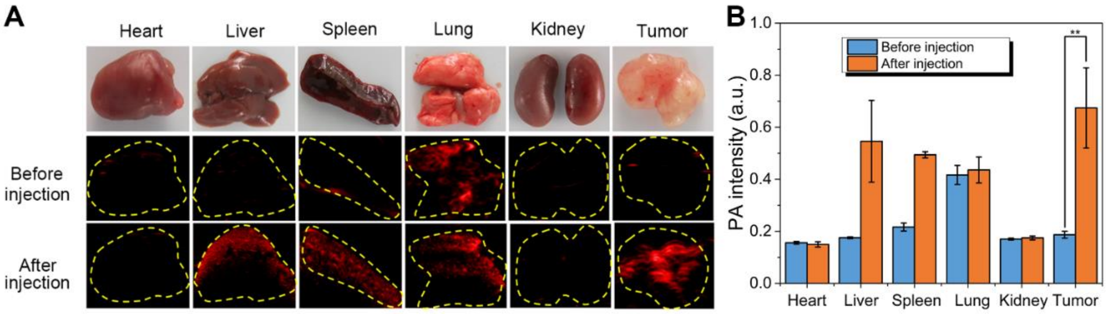

Figure S7. *Ex vivo* PA intensity in various tissues from xenografted BALB/c nude mice before/after injection with PDPP3T@PNIPAMAA IPNs (**p<0.01).

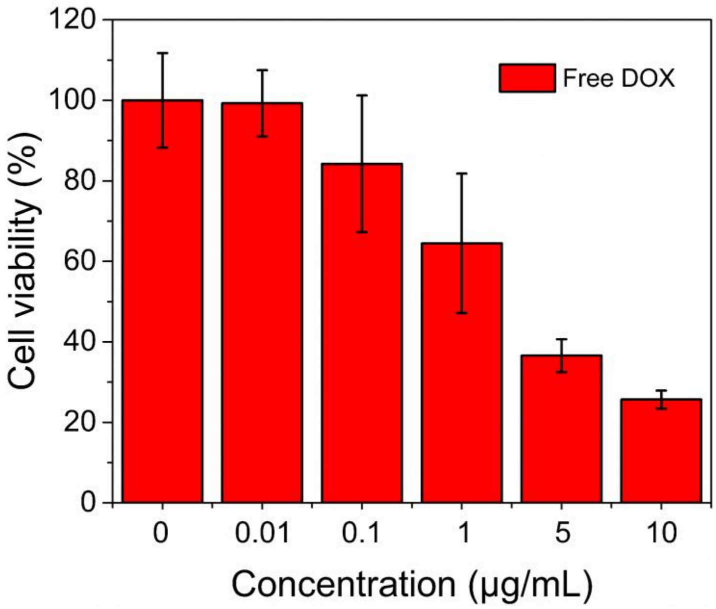

Figure S8. Cytotoxicity of HeLa cells evaluated by MTT assay after incubated with free DOX.

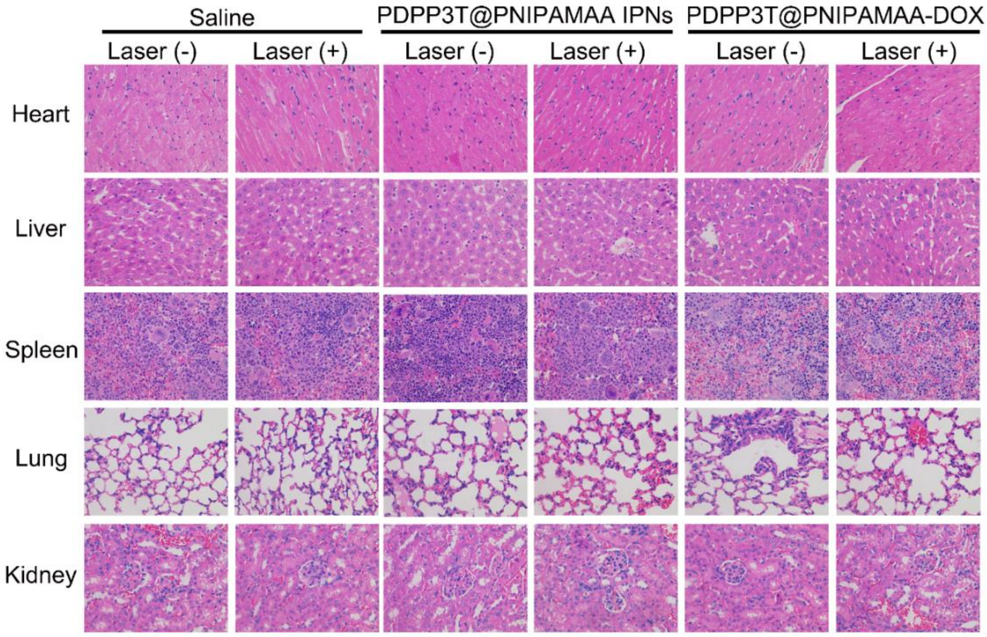

Figure S9. H&E analyses of major organ tissues.
